# The Application of Adeno-Associated Viral Vector Gene Therapy to the Treatment of Fragile X Syndrome

**DOI:** 10.3390/brainsci9020032

**Published:** 2019-02-02

**Authors:** David R. Hampson, Alexander W. M. Hooper, Yosuke Niibori

**Affiliations:** Department of Pharmaceutical Sciences, Leslie Dan Faculty of Pharmacy, University of Toronto, Toronto, ON M5S 3M2, Canada; alex.hooper@utoronto.ca (A.W.M.H.); yo.niibori@gmail.com (Y.N.)

**Keywords:** adeno-associated virus, autism spectrum disorders, cerebral spinal fluid, fragile X mental retardation protein, neurodevelopmental disorders, viral vector

## Abstract

Viral vector-mediated gene therapy has grown by leaps and bounds over the past several years. Although the reasons for this progress are varied, a deeper understanding of the basic biology of the viruses, the identification of new and improved versions of viral vectors, and simply the vast experience gained by extensive testing in both animal models of disease and in clinical trials, have been key factors. Several studies have investigated the efficacy of adeno-associated viral (AAV) vectors in the mouse model of fragile X syndrome where AAVs have been used to express fragile X mental retardation protein (FMRP), which is missing or highly reduced in the disorder. These studies have demonstrated a range of efficacies in different tests from full correction, to partial rescue, to no effect. Here we provide a backdrop of recent advances in AAV gene therapy as applied to central nervous system disorders, outline the salient features of the fragile X studies, and discuss several key issues for moving forward. Collectively, the findings to date from the mouse studies on fragile X syndrome, and data from clinical trials testing AAVs in other neurological conditions, indicate that AAV-mediated gene therapy could be a viable strategy for treating fragile X syndrome.

## 1. Molecular and Clinical Aspects of FXS

Fragile X syndrome (FXS) is a genetic disorder caused by a pathological expansion of a triplet repeat in the 5’ untranslated region of the FMR1 gene. Expansion from the normal 5–55 repeats to 200 or more causes hypermethylation of the gene promoter and shutdown of transcription and translation of the encoded protein, fragile X mental retardation protein (FMRP) [1,2]. The expanded repeat also induces formation of RNA:DNA duplexes that induce epigenetic gene silencing [3]. Although most persons with FXS do not express FMRP, some individuals with the full mutation do produce low amounts of the protein (<10% of “normal levels”). In persons without FXS, the levels of FMRP in human brain [4], and in human blood platelets, a rich source of FMRP [5], vary over a wide range, and interestingly in persons with FXS, FMRP levels have been shown to positively correlate with intelligence scores [5,6]. As delineated below, these findings have implications for FXS gene therapy. Individuals with an intermediate triplet expansion of about 60–199 repeats, called the “premutation”, are at risk for developing adult onset fragile X-associated tremor/ataxia syndrome (FXTAS) or premature ovarian insufficiency [7]. Fragile X-associated tremor/ataxia syndrome is a late-onset neurodegenerative condition that manifests in some carriers of the *FMR1* premutation; approximately 40–75% of males and 16–20% of females with a premutation develop FXTAS [8]. 

FMRP is a master regulator of gene expression in various organs including the brain, testes, and ovaries where it is highly expressed. The *FMR1* gene undergoes alternative splicing to generate at least 16 mRNA isoforms [9]. FMRP is a pleiotropic protein that plays a critical role in both CNS development and oogenesis [10,11], and in cognitive function in the mature brain as demonstrated in a fragile X conditional restoration mouse line [12]. FMRP contains several mRNA binding motifs that are capable of binding hundreds of mRNAs [13,14]. However, the ability to bind hundreds of mRNA substrates has been questioned by some who instead suggest that the protein regulates only a restricted number of key mRNA substrates including diacylglycerol kinase kappa, which is thought to act as a master regulator that controls switching between the diacylglycerol and phosphatidic acid signaling pathways [15].

In addition to its mRNA binding role, FMRP also associates with and regulates other proteins directly. Salient examples include voltage-gated potassium channels which modulate the sodium-dependent action potential in neurons [16,17,18]. In the case of the Kv1.2 potassium channel, it has been demonstrated using wild-type and *Fmr1* KO mice, that FMRP plays an essential role in cerebellar inhibitory interneurons by both assisting in the trafficking of the channel to nerve terminals, and by facilitating Kv1.2 channel activity. In cerebellar basket cell interneurons, Kv1.2 controls (inhibits) gamma-aminobutyric acid (GABA) release from basket cell terminals [18]. Therefore, the absence of FMRP at the cerebellar basket cell-Purkinje neuron synapses results in elevated GABA release onto Purkinje dendrites causing a reduction in Purkinje neuron activity. In addition to the Fmr1 KO mouse, reduced Purkinje neuron activity has been observed in other mouse models of autism; therefore, we speculated that this property might be a common denominator in mediating some features of the autistic phenotype [18].

The clinical profile of FXS overlaps with that of autism spectrum disorders (ASD). Shared characteristics include impaired communication, sensory hypersensitivity, anxiety, stereotyped or repetitive behaviors, aggression, and cognitive impairment [19]. However, not all persons with FXS meet the clinical diagnostic criteria for ASD; approximately 50% of males and 20% of females with FXS meet the criteria for autism [20]. As seen in various forms of ASD, individuals with FXS are at increased risk for developing epileptic seizures in childhood. It has been estimated that about 12% of males and 6% of females with FXS experience spontaneous seizures during early childhood [19,21]. The seizures are partial complex, generalized tonic-clonic, and/or absence seizures that typically resolve by puberty.

Current pharmacotherapy for FXS still consists exclusively of symptomatic drug treatment. Examples include stimulants, antidepressants, antipsychotics, and valproate that are each somewhat effective in suppressing a subset of symptoms [22]. Over the past decade more than a dozen clinical trials of compounds considered to be potential second generation drug candidates were conducted based on what was thought to be mechanisms more closely linked to the underlying dysfunctional neurochemical pathways in the disorder. The most prominent examples include metabotropic glutamate receptor 5 antagonists and an agonist at the GABA_B_ receptor. Progression to Phase 2 (mGluR5 antagonists) and Phase 3 (a GABA_B_ agonist) clinical testing was based on extensive encouraging results from both in vitro tests, and in vivo animal analyses using the fragile X knockout mouse model. However, none of the clinical trials led to new drug approvals due in large part to lack of efficacy [23]. 

The failure of small molecule drugs in clinical trials to date might reflect, in part, the pleiotropic nature of FMRP. In light of the many varied roles of FMRP it should not be completely surprising that drugs that specifically block or activate individual receptors, enzymes, or other proteins may not be sufficient to provide a deeper and more comprehensive correction of the disorder. *A priori*, a major motivation for pursuing small molecule drugs has been based on the idea that a particular receptor, enzyme, or other type of protein that is over- or under-expressed, or is over- or under-active, induces a major symptom or cluster of symptoms, and that when normalized, will result in major therapeutic improvements. The fundamental issue here stems from the difficulty in determining which of the many potential targets of FMRP, when corrected, will actually result in robust, measurable improvements in physiology, behavior, and health of persons with FXS.

An alternative approach is to try to correct the basic underlying biochemical defect of the disorder-the absent or dramatically reduced levels of FMRP in the brain. Viral vector-mediated gene therapy is one potential avenue for rectifying the fundamental molecular defect in FXS. This approach may also be amenable to other genetic disorders associated with ASDs [24]. The essence of the strategy is simple - to incorporate the coding sequence of FMRP, along with appropriate regulatory elements, into the genome of a recombinant viral vector so as to provide a vehicle for transducing (expressing) the recombinant FMRP “transgene” protein in brain cells [22]. However, as outlined below, achieving this to obtain substantial therapeutic improvement and correction of FXS is complicated and challenging from several perspectives. 

## 2. General Features of Adeno-Associated Viral Vectors Used as Gene Therapy Vehicles

Recombinant adeno-associated viral (AAV) vectors are currently the most widely employed class of viral vectors for gene therapy. Less commonly used vectors include adenovirus vectors and lentivirus-based vectors. The lentiviral vectors have been used successfully in ex vivo treatments, for example in treating X-linked cerebral adrenoleukodystrophy where therapy requires infusion of purified autologous CD34+ cells transduced with a lentiviral vector [25]. Nevertheless, AAV vectors have advantages over other types of vectors for gene therapy. In addition to not causing any known pathology, additional upsides of the AAV class of vectors include high infectivity of cells and tissues, small particle size (about 25 nanometers in diameter) facilitating diffusion through tissues, multiple unmodified natural serotypes and modified synthetic serotypes encoding viral capsid proteins, non-replicating, low (but not zero under some conditions) genomic DNA integration, and relatively low immunogenicity [26]. 

Limitations of AAV vectors are the restricted room for target DNA insertion into the AAV vector and the presence of pre-existing circulating anti-AAV capsid antibodies in up to about half of the human population [27,28]. Preexisting neutralizing anti-AAV antibodies present in the body prior to gene therapy administration can reduce therapeutic efficacy, while additional antibody and T cell induction after AAV vector treatment can further impair efficacy. Obviously, this also presents a problem for a second injection of AAV in cases where a boost in the transgene expression level is desirable.

In addition to multiple serotypes, recombinant AAV vectors are of two main types; single-stranded vectors (ssAAV) and self-complementary vectors (scAAV). The former possesses a larger insertion capacity of about 4.6 kilobases of DNA, while the latter has a very limited capacity restricted to a maximum of 2.4 kilobases, but is more efficient at expressing the inserted transgene [26]. It should be noted that these size limits must include not only the coding sequence of the desired transgene but also the regulatory elements (e.g., promoter) and other regulatory sequences such as the short but mandatory inverted terminal repeats required in all AAV vectors. In the context of the *FMR1* gene sequences, ssAAV vectors are deployable for all isoforms including the full-length isoform 1 [29,30]. The scAAV vectors may also be amenable for use with the shorter isoforms and possibly isoform 1 depending on the size of the promoter used. Another potential issue with AAVs is the possibility of insertional genotoxicity leading to oncogenesis, particularly in the liver, where AAV9 encapsulated genome capsids have been shown to integrate under certain conditions, for example when a strong ubiquitous promoter is used to drive expression [31]. So far, this phenomenon has only been reported in mice, and whether or not AAV vector incorporation into human genomic DNA occurs, and whether it presents a genotoxic threat in humans remains unknown [32].

Recombinant AAV vectors used for gene therapy are packaged into virus particles and subjected to purification by density gradient centrifugation (e.g., with iodixanol) followed by high performance liquid chromatography with ion exchange chromatography. After binding to an AAV receptor protein on the surface of cells, the viral particle enters the cell and begins to transcribe and translate copies of the encoded transgene, but the virus itself does not replicate. Each AAV serotype has one or more receptor protein(s) that is primarily responsible for mediating viral uptake into cells and tissues [28]. The choice of AAV serotype is important as the available array of virus serotypes have different but partially overlapping tissue and cellular preferences. For CNS applications, AAV9 has been the most widely studied in animal studies as it has an excellent ability to transduce neurons and glia. In addition to AAV9, AAV2 and AAVrh10 have also been used to express proteins in the brain in clinical trials [33]. The development of novel modified AAV serotypes is an active area of research with the goal of discovering improved versions of AAVs that possess higher selectivity and binding to more restricted subsets of cells and tissues. 

In addition to relatively low tissue and cell-type selectivity imparted by most of the natural viral serotypes, further restriction of transgene expression is achieved via the use of customized gene promoters. Several CNS specific or selective promoters have been tested in AAV gene therapy experiments in preclinical animal studies. Examples include the synapsin promoter for neuron-selective expression [29], the dlx5/6 promoter for GABAergic inhibitory neurons [34], glia fibrillary acidic protein for expression in astrocytes [35], and myelin basic protein for expression in oligodendroglia [35]. Further refinement of short, cell-type specific gene promoters for use in AAV gene therapy will likely be actively pursued over the next few years.

Another very attractive aspect of AAV vectors for treating CNS disorders is the capacity for long-term expression of the therapeutic protein. Upon administration, recombinant AAV-mediated therapeutic protein expression gradually ramps up over the first few weeks after injection and plateaus about 3–4 weeks post-injection. The epichromosomal presence of the AAV is static and can translate into long-term expression of the desired transgene. In tissues containing dividing cells with cellular turnover, expression levels will dwindle over time. In non-dividing long-living cells like differentiated neurons, recombinant transgenes are typically expressed for years. For example, transgenes expressed from injected AAVs have been shown to persist in the primate brain and maintain a therapeutic effect for up to 10 years [36,37,38]. This is a crucial aspect of CNS gene therapy considering the invasive nature of the treatment when administered into the CNS via direct parenchymal injections or into the cerebral spinal fluid (CSF). 

## 3. Theoretical Aspects of Treating FXS with Viral Vector-Mediated Gene Therapy

As noted above, developing AAV-FMRP gene therapy for FXS presents a numbers of issues that need to be resolved for moving this potential biological therapeutic drug from the laboratory to clinical trials and beyond. At the top of the list are issues associated with CNS delivery of biologically-based therapeutic drugs such as viral vectors. Because of the pan distribution of FMRP throughout the brain, direct injections into the parenchyma are largely precluded due to lack of sufficient spread of the virus to other brain regions. The logical solution would be to administer the vector systemically, for example, via intravenous injection. Several obvious problems here include the very large quantities of a very expensive vector that need to be injected, much or most of which binds to and is taken up by peripheral organs such as the liver and heart that act as sinks for several types of viral vectors, and the potential complications of virus-induced side effects from treating patients with the large doses that would be required to obtain adequate FMRP expression levels in the CNS [39]. 

A potential solution is to infuse the vector into the cerebral spinal fluid (CSF) through the spinal canal (intrathecal injection, i.t.), by intra-cisterna magna (i.c.m.) injection near the base of the skull, or by intracerebral ventricular injection (i.c.v.). All three of these routes are applicable to studies in experimental mammals, while i.t. injections have been most the widely used mode for administering drugs into the CSF in humans and non-human primates [40,41,42,43]. However, i.c.m. infusions are also feasible and may become more widely used in clinical testing. An important parameter after injection into the CSF is the extent of diffusion of the vector from the point(s) of injection. It has been suggested that the flow of CSF through the so-called “glymphatic system” of the brain may facilitate dispersion of injected AAV particles [29]. For treatment of FXS, the goal is to mimic as closely as possible the natural brain-wide expression of FMRP. As discussed in Arsenault et al., 2016 [30], i.c.v. injection of AAV-FMRP in mice at Postnatal Day 2 gave a wider brain distribution of the vector than injection at Postnatal Day 5. This might be related, in part, to the less mature ependymal lining surrounding the walls of the ventricles in the mouse brain two days after birth compared with a few days later. Because the very early postnatal rodent brain corresponds approximately to a third trimester human fetus, vector injection into the brain of a rodent immediately after birth is not directly translatable to the situation for human therapeutics. Whether or not the drop off of vector diffusion seen in mice occurs in other mammalian species has not been sufficiently explored to date, but such experiments conducted in other species, including non-human primates, would be of considerable value.

A second issue that must be dealt with is proper dosing with the goal of achieving normal “wild-type” levels of FMRP in as much of the brain as possible. Achieving vector-derived FMRP expression at an adequate level in the brain, and in the appropriate types of cells are important parameters that are discussed further below. Viral vectors differ from small molecule drugs in that they are typically given only once or a few times, and are not easily amenable to dose modulation. This means that the injected dose(s) must be carefully established prior to initiation of treatment because with current vector systems, there is no opportunity for reducing the dosage and level of transgene expression downward. Additional dosing to achieve a higher level of expression can be accomplished by one or more subsequent treatments, but as noted above this comes with increased risk of inducing the immune system to generate anti-capsid antibodies that can neutralize or hinder the efficacy of the viral vector therapeutic effects (see Reference [28] for discussion of this topic). 

Cell-type selectivity is another variable that needs to be considered for CNS treatment with viral vectors and represents a strength of this approach. Unlike many small molecule CNS drugs which generally lack cell-type targeting specificity, viral vectors capitalize on the use of selective promoters to direct transgene expression in subtypes of neurons and glia. In the case of FXS, selection of a suitable promoter to use is somewhat complicated by the observation that in mice, early in postnatal brain development, FMRP is expressed in virtually all types of neurons and glia. Over the first four weeks after birth, with several exceptions such as the corpus callosum, glial expression gradually down-regulates so that in the mature brain FMRP expression in glia is very low relative to the moderate to high expression in neurons [44]. Extensive analyses of FMR1 gene regulatory elements have been carried out, and although a minimal FMR1 promoter region that works in cells in vitro has been identified, it is not known if additional regulatory elements might be necessary for proper neuronal regulation of the FMR1 gene in vivo [45,46,47,48,49,50,51]. Therefore, due to the size constraints of some viral vectors, and the fact that a compact “mini” version of the Fmr1 promoter has not been identified for use in vivo, other mini-gene promoters have been tested in gene therapy experiments conducted in the Fmr1 KO mouse.

## 4. Successes and Shortcomings of AAV-Mediated Gene Therapy Studies Conducted in the *Fmr1* Knockout Mouse

To date, studies using viral vector gene therapy in the *Fmr1* KO mouse model of FXS have employed AAV5 and AAV9 vectors and the coding sequence of the full-length *Fmr1* isoform 1. The first published report on the testing of a viral vector for FXS was by Zeier et al. (2009) [52]. This group used an AAV5 vector with a chicken β-actin core promoter containing elements from the cytomegalovirus immediate-early enhancer, the full-length *Fmr1* coding sequence, and a FLAG epitope tag. The virus was injected bilaterally directly into the hippocampus of 5 week-old *Fmr1* KO mice. At the cellular level, *Fmr1* KO mice have been shown to display abnormal synaptic plasticity as manifested by impaired long-term potentiation and enhanced long-term depression. Zeier et al. (2009) conducted brain slice recordings of extracellular postsynaptic field potentials from area CA1 of the hippocampus 3–5 weeks post-injection [52]. *Fmr1* KO mice treated with AAV5-FMRP showed correction of abnormally enhanced long-term depression. This finding is important because altered synaptic plasticity in FXS is thought to contribute to the cognitive deficits in the disorder.

The next published study (Gholizadeh et al., 2014) was conducted by our group which utilized an AAV9 vector administered by i.c.v. injection at Postnatal Day 5 [29]. Here, a series of biochemical and behavioral tests was used to assess the degree of reversal of abnormal endophenotypes at four to eight weeks of age. The AAV vector contained the full-length mouse *Fmr1* isoform 1 under the control of the neuron-specific synapsin promoter. Anatomical and cellular analyses using immunocytochemistry revealed transgene expression in neurons in various forebrain regions with lower expression in more caudal areas of the CNS. The observed rostral-to-caudal gradient was expected based of the location of the injection site in the lateral ventricles. Quantitative western blotting using an anti-FMRP antibody of samples collected at Postnatal Days 30 to 60 showed a range of transgene expression levels across different brain regions, from a high of 71% in the cerebral cortex to a low of 18% in the striatum compared to baseline wild-type expression. Low but detectable levels were also seen in the cerebellum. AAV-FMRP expression was still detected at 7-months post-injection demonstrating the stability of the FMRP transgene.

For behavioral analyses, a range of tasks were employed to judge normalization after AAV-FMRP [29]. In uninjected *Fmr1* KO mice, motor activity is increased relative to wild-type mice, marble burying reflecting repetitive behavior, is increased, social dominance as determined by the tube test is decreased, as is the number of ultrasonic vocalizations. Treatment of KO mice with AAV-FMRP normalized stereotyped behavior and social dominance, whereas no significant changes were observed in motor activity or the frequency of vocalizations. *Fmr1* KOs but not wild-type mice are highly susceptible to sound-induced (audiogenic) seizures. This is a dramatic and robust endophenotype of Fmr1 mice in which typically 50–75% of the tested KO mice undergo seizures [53,54]. Gholizadeh et al., 2014 reported that the incidence of audiogenic seizures in both PBS-injected Fmr1 KO mice and AAV-FMRP-injected mice was significantly elevated compared to PBS-injected wild-type mice, however, the AAV–FMRP group was not different to the PBS-injected Fmr1 group.

To summarize, the Gholizadeh et al. (2014) study injected AAV9-FMRP with a neuron-specific promoter into the CSF of *Fmr1* KO mouse neonates and obtained FMRP expression mainly in forebrain regions; this translated into correction of repetitive behavior and social dominance but not motor hyperactivity, abnormal calling frequency, or increased seizure susceptibility [29].

The third published study to date was also carried out by our group. In the Arsenault et al. (2016) study we again used AAV9 and mouse isoform 1 driven by neuron-specific promoters, but instead of injecting control mice with phosphate-buffered saline, an AAV9 carrying no transgene was used as the negative control (termed AAV empty vector or “AAV-EV” here and “AAV-null” in Arsenault et al., 2016) [30]. This study had several new objectives. First, we compared FMRP transgene dispersion in the brain after i.c.v. injection on Postnatal Days 1, 2, 3, or 5. It was concluded that treatment on Postnatal Day 2 or 3 gave the best results with respect to the health of the mice, spread of the vector within the brain, transgene expression levels, and therapeutic outcome. It should be noted however that this early postnatal age in mice corresponds to roughly the third trimester of a human fetus, and therefore, strictly in terms of the timing, is not directly translatable to a human therapeutic situation (see additional discussion on this topic below). 

A second objective was to extend behavioral testing to additional tasks not carried out in the previous Gholizadeh et al. (2014) study [29]. The new tasks included the elevated plus maze, ostensibly measuring “anxiety”, and prepulse inhibition together with the acoustic startle response to measure auditory sensory perception. AAV-FMRP corrected the abnormal “reduced anxiety” and the elevated acoustic startle response observed in the control KO mice injected with AAV-EV. Interestingly, on the elevated plus maze task, we (Arsenault et al., 2016) [30], and others have shown that *Fmr1* KO mice display an increase in the number entries and the time spent in the open arms of the maze [55,56,57]. This phenomenon has also been described for other mouse models of autism spectrum disorders [58,59]. Traditionally, more entries into and more time spent in the open arms are interpreted as a reduction in anxiety. This is perplexing as it is well established that persons with FXS and ASD typically display high anxiety. Arsenault et al. (2016) offered an alternate interpretation whereby rather than measuring anxiety, performance on the elevated plus maze test in *Fmr1* KO mice, and perhaps other lines of mice with autistic features, may instead reflect cognitive impairment [30]. In this interpretation, cognitively impaired mice, unlike wild-type mice, are not cognizant of the potential danger of spending time in open spaces. The inability to appreciate potentially dangerous situations may be a trait characteristic of some and possibly many persons with autism (see References [60,61]). 

A third objective of the Arsenault et al. (2016) study was to examine the normalization of FMRP substrates in the CNS after treatment [30]. The highly abundant post-synaptic density protein 95 (PSD-95), a scaffolding protein required for synaptic function and plasticity, is known to be down-regulated in *Fmr1* KO mice. We also ascertained that the transcription modulator MeCP2, the mutation of which causes Rett Syndrome and is associated with some autistic features, is a substrate for FMRP (i.e., MeCP2 mRNA binds to FMRP). We found that MeCP2 protein levels are elevated in the *Fmr1* KO mouse brain. Both PSD-95 and MeCP2 proteins reverted to wild-type levels 4 weeks after injection with AAV-FMRP under the control of the synapsin promoter. Therefore, at the biochemical level, treatment with AAV-FMRP was capable of correcting the expression of key proteins that are regulated by FMRP.

Finally, the fourth major objective was to probe the consequences of under and over-expression of FMRP relative to normal wild-type levels in the CNS. The purpose of this part of the study was to conduct the first dose ranging study of FMRP in *Fmr1* KO mice. We compared five treatment groups injected with one of three test vectors: wild-type (WT) mice treated with AAV-EV (the control baseline group), WT mice treated with the AAV-FMRP vector, and *Fmr1* mice treated with AAV-EV (control baseline group of impaired KO mice), AAV-FMRP (the therapeutic treatment group), or the AAV-FMRP high-expressing vector representing a high over-expression group. This allowed for an assessment of the effects of a wide range of FMRP levels in the brain. Distillation of the results indicated that partial rescue of the *Fmr1* phenotype was observed in mice with forebrain FMRP levels of approximately 35–70% of wild-type, while moderate over-expression of up to approximately 120–140% did not induce behavioral abnormalities. However, massive over-expression of 200–600% of normal wild-type levels induced aberrant behaviors such as hyperactivity and an abnormally reduced startle response. The take-home message here is that modest under-expression appears to produce some therapeutic effects, while modest over-expression of FMRP does not appear to induce pathology. These findings suggest that a fairly wide range of CNS FMRP levels could translate into therapeutic benefits. 

## 5. The Pathway from Preclinical Experimentation to a Clinical Trial

The three studies reviewed above that explored the efficacy of AAV-FMRP therapy in the mouse KO model have collectively demonstrated a general proof-of-principle whereby AAV-FMRP fully or partially corrected abnormalities on several behavioral tasks and in vitro assays conducted at the cellular and biochemical levels. However, considering the high bar likely to be set by regulatory agencies for moving forward with a Phase 1 clinical trial for FXS, additional evidence supporting the efficacy and safety of viral vector-mediated gene therapy would bolster the case for proceeding to a clinical trial. To date, clinical trials for CNS disorders using viral vector-mediated gene transfer were approved by regulatory agencies, in part, because they addressed unmet needs for treating severe degenerative disorders such as Parkinson’s and Alzheimer’s disease, or neurodevelopmental disorders that cause paralysis and/or death such as spinal muscular atrophy type 1 and giant axon neuropathy. In contrast, FXS and most other forms of autism spectrum disorders do not lead to dementia, severe paralysis, or premature death. Nevertheless, FXS and ASDs are life-long disorders in which the majority of affected individuals display moderate to severe cognitive impairment along with a slew of additional medical problems. Most will need some type of life-long care from an early age. Therefore, the discovery and development of an effective treatment would have enormous benefits not only for those with FXS, but also their families and health care funders. 

**Figure 1:**
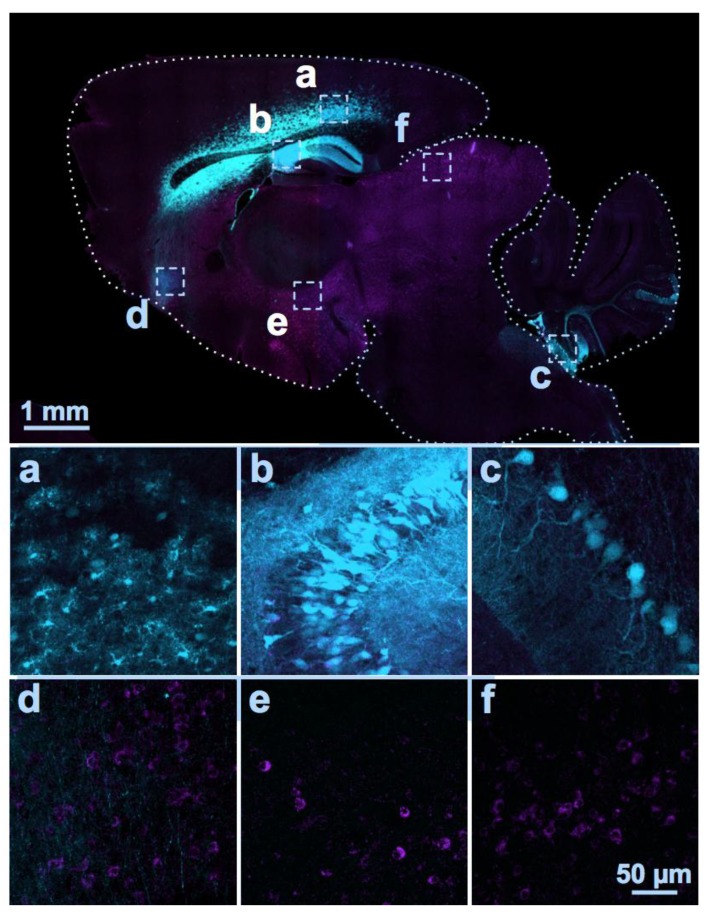
Distribution of AAV serotype 9-mediated transgenes following an i.c.v. and i.c.m. double injection. Two different AAV vectors were injected into a C57BL/6J mouse at Postnatal Day 2. One encoding a cytomegalovirus promoter driving enhanced green fluorescent protein (eGFP; visible in cyan color; dose: 2 × 10^10^ GC) was injected bilaterally into the lateral ventricles (i.c.v.); the other using a GABAergic neuron promoter driving a myc-tagged sodium channel protein was injected into the cisterna magna (i.c.m., visible as magenta color; dose: 3 × 10^10^ GC). At Postnatal Day 18, the distributions of the two proteins were examined in sagittal sections of fixed brain tissue using immunocytochemistry and anti-c-myc and anti-green fluorescent protein (GFP) antibodies. GFP immunolabeling was detected in the cerebral cortex (**a**), hippocampus (**b**), and Lobules VII to X of the cerebellum (**c**), while the myc-tagged protein was expressed in soma and eGFP was expressed in axons in the frontal regions of the brain (**d**), hypothalamus, (**e**), and the inferior colliculus (**f**). Thus, the two vectors administered into the CSF at the same time by two different injection routes were largely distributed in distinct brain regions.

Optimization of several technical parameters should also be considered in moving forward towards clinical testing. First, further assessment of drug delivery methods could be beneficial. Ultimately, experimental treatment methods in the laboratory should be representative of routes of administration that will be used in patients. Assessing additional drug delivery protocols that could provide more wide-spread diffusion of the AAV vectors after injection into the CSF, especially to more caudal regions of the brain, would likely boost the therapeutic response. Simply administering a larger amount of the virus from a single point-source injection may not be a solution because of the liability of transgene over-expression near the needle injection site. However, injecting at more than one site might provide a work-around. Our preliminary observations in mouse brain suggest that simultaneously injecting the vector into the CSF at more than one site, for example, an i.c.v. injection together with an i.c.m. (or i.t.) injection, may enhance transgene coverage in the brain (e.g., see Figure 1). 

The small size of the mouse brain relative to the human brain is a limitation of mouse models. However, previous studies in dogs, pigs, and monkeys have demonstrated successful scale up of AAVs injected into the CSF [62,63,64]. So far, in human trials testing AAVs in neurological disorders, direct parenchymal injections have been the most widely used, while a few have utilized i.t. or intravenous treatment [33,65]. In the highly successful AAV trial of spinal muscular atrophy type I, the pediatric subjects were injected intravenously with a high dose of an AAV9 therapeutic vector [66]. This is now being followed up by a second Phase 1 trial in which the vector is administered i.t. [32]. Although intravenous AAV administration is attractive from the standpoint of likely attaining a more thorough and uniform coverage of the brain, the downsides include the potential inability to reach a sufficiently high level of transgene expression in the brain to produce a therapeutic effect, the as-yet unclear medical consequences of high systemic dosing with an AAV, and the substantially higher cost of treating patients with large amounts of a highly purified virus.

Second, testing additional isoforms of FMRP could be informative. The human *FMR1* gene on chromosome Xq27.3 is 38 kilobases long and encompasses 17 exons and 16 introns. As outlined above, the three studies on AAV-FMRP published to date have all used the full-length mouse isoform 1 encompassing 4411 nucleotides. This was based on the assumption that the full-length isoform 1 was a major variant. However, this turned out to be incorrect as *FMR1* mRNA isoform expression surveys conducted in human [67,68] and mouse blood cells and brain tissue [69] have indicated that isoform 1 is expressed at lower levels compared to several other splice variants. For example, isoforms lacking exon 12 appear to be among the most abundant [68]. Moreover, deletion of exon 14 has been found to affect the subcellular localization of FMRP [67,70], and in premutation carriers all isoforms are elevated with isoforms 10 and 10b showing the largest increase; the consequences of differential elevations in *Fmr1* isoforms remain unknown [9]. It is important to note that no studies have yet examined the translated FMRP protein isoforms in terms of functional differences or even relative levels of expression in the mammalian brain. This could be important because mRNA levels do not always accurately reflect expression levels of the encoded proteins.

Third, in addition to ongoing work in mice, testing AAV-FMRP distribution and efficacy in additional species could provide further confidence in establishing AAV-FMRP as a viable therapeutic agent. In terms of an alternative disease model, the logical choice would be the *Fmr1* KO rat. Although the *Fmr1* KO mouse has been the most widely used animal model of FXS, it does display somewhat subtle and difficult to replicate endophenotypes on some behavioral tests, especially those tasks probing cognitive function. Although the adult rat brain is only about four times larger than a mouse brain (about 2 g vs. 0.5 g), rats are generally thought to be smarter and capable of performing more difficult cognitive tasks [71]. Deficiencies with other mouse models of CNS disorders have also prompted the neuroscience research community to pursue the development of rat disease models, for example Huntington’s Disease, Rett Syndrome, and Parkinson’s Disease [72,73,74]. 

Because it is much newer on the scene, the *Fmr1* KO rat is relatively uncharacterized compared to the *Fmr1* KO mouse. Nevertheless, the *Fmr1* KO rat has been reported to display impairments or abnormalities on tests measuring sensory perception, communication, cognition, and other functions [75,76,77,78,79,80,81]. The first version of the KO rat model was created in collaboration with Autism Speaks in the U.S.A. and underwent initial phenotypic characterization by Paylor and colleagues at Baylor College of Medicine [75]. Juvenile animals exhibited abnormalities in ASD phenotypes including juvenile play, perseverative behaviors, and sensorimotor gating. Further early study of this line showed cortical representation of speech sounds is impaired in *Fmr1* KO rats, despite normal speech discrimination behavior. Evoked potentials and spiking activity in response to speech sounds, noise burst trains, and tones were degraded in primary auditory cortex and the anterior and ventral auditory fields compared to wild-type rats [76].

Abnormalities were also documented in an analysis of neuronal morphology and neurochemistry in the auditory brainstem of *Fmr1* knockout rats [78], and importantly, KO rats have been reported to have deficits in hippocampal-dependent, but not hippocampal-independent memory, indicating that the absence of FMRP causes defects in episodic-like memory [77]. Robust changes were also reported in long-term potentiation, long-term depression, and in hippocampal-dependent memory as assessed using the Morris water maze [80]. Finally, cortical electroencephalographic (EEG) recordings conducted on juvenile *Fmr1* KO rats showed that during quiet rest when activity in wild-type rats became dominated by the inactivated state (3–9 Hz), cortical activity in the KO rats remained activated, resulting in increased high-frequency and reduced low-frequency power during rest [79]. Moreover, firing rate correlations revealed reduced synchronization in *Fmr1* KO rats, particularly between fast-spiking inhibitory interneurons. The findings from this study, together with data from EEG analyses conducted in *Fmr1* KO mice [82], and from clinical EEG studies in subjects with FXS [83], are important because (a) they provide new insight into cortical defects in the disorder, and (b) indicate that EEG may be a very useful tool as an endpoint measurement in clinical trials for FXS. Taken together, these findings suggest that the *Fmr1* KO rat is likely to be a useful addition for testing AAV-FMRP efficacy, particularly in probing cognitive and sensory functions.

## 6. Conclusions

Gene therapy studies conducted so far have used the mouse model of FXS and demonstrated full or partial correction of selected deficits after early postnatal treatment with AAV-FMRP. Two of the published studies injected AAV-FMRP vectors containing neuron-specific promoters into the CSF of the neonatal mouse brain to drive FMRP transgene expression in neurons [29,30]. The lower levels of expression of FMRP in more caudal regions of the brain, such as the cerebellum and brainstem compared to forebrain regions after ventricular injection, may explain in part, the lack of a more complete correction of the phenotype in the *Fmr1* KO mouse. Whether or not this is an issue for the treatment of human subjects remains unknown. Injecting AAV-FMRP via the intra-cisterna magna route with (Figure 1) or without the simultaneous injection into the ventricles may improve brain coverage and therapeutic efficacy. Although systemic injection (e.g., i.v.) of AAV-FMRP has not been assessed in rodent models of FXS, translating this to human therapy faces the issues of high peripheral organ uptake of the vector, possible elevated immune reactions, and the very high cost of the large dose that would be required for systemic administration. Finally, in terms of the effects of varying levels of FMRP expression, the findings from the Arsenault et al. (2016) study suggest that FMRP transgene expression below wild-type levels may be sufficient to produce at least some therapeutic benefits, and conversely, mild over-expression does not appear to be associated with deleterious consequences [30]. 

Moving forward, useful experimentation should encompass exploring the therapeutic effects of administering AAV-FMRP at later time points after birth to more closely mimic a clinical trial situation. Additionally, results from the study of *Fmr1* conditional knockout and conditional restoration mice have demonstrated that the selective deletion of FMRP in glia or in the prefrontal cortex after birth results in cellular and behavioral deficits [12,84]. The observation that behavioral deficits induced by the deletion of FMRP in the prefrontal cortex during development could then be reversed in the same line of mice after FMRP expression in the adult cortex suggests that (a) the continuous expression of FMRP is needed in the mature CNS for normal brain function, and (b) that viral vector-mediated production of FMRP initiated in adult or young adults might be capable of rescuing at least some aspects of impaired brain function in FXS.

Additionally, more in-depth characterization of rodent tests that are translatable to a clinical trial, such as prepulse inhibition and EEG, and the effects of AAV-FMRP on normalization of these parameters, could prove essential in eventual approval of this experimental biological therapeutic drug. Finally, encouraging positive results from recent clinical trials using AAVs in severe childhood diseases have generated extremely useful information from several technical standpoints ranging from drug delivery and drug dosage to post-treatment care and follow-up. Overall, the momentum attained in the field of viral vector-mediated gene therapy has now created further incentive to continue to develop this technology for treating not only FXS, but also other neurodevelopmental disorders caused by known gene mutations.
